# Bacteriophages and antibiotic interactions in clinical practice: what we have learned so far

**DOI:** 10.1186/s12929-022-00806-1

**Published:** 2022-03-30

**Authors:** Marzanna Łusiak-Szelachowska, Ryszard Międzybrodzki, Zuzanna Drulis-Kawa, Kathryn Cater, Petar Knežević, Cyprian Winogradow, Karolina Amaro, Ewa Jończyk-Matysiak, Beata Weber-Dąbrowska, Justyna Rękas, Andrzej Górski

**Affiliations:** 1 Bacteriophage Laboratory, Hirszfeld Institute of Immunology and Experimental Therapy, Polish Academy of Sciences, 53-114 Wrocław, Poland; 2 Phage Therapy Unit, Medical Center of the Hirszfeld Institute of Immunology and Experimental Therapy, Polish Academy of Sciences, 53-114 Wrocław, Poland; 3 Department of Clinical Immunology, Transplantation Institute, Medical University of Warsaw, 02-006 Warsaw, Poland; 4 Department of Pathogen Biology and Immunology, University of Wrocław, 51-148 Wrocław, Poland; 5 Rush University Medical Center, 1620 W. Harrison St., Chicago, IL 60612 USA; 6 Department of Biology and Ecology, Faculty of Sciences, University of Novi Sad, 21000 Novi Sad, Republic of Serbia; 7 Faculty of Life Sciences, University College London, London, WC1E 6BT UK; 8 Polpharma Biologics S. A., 80-172 Gdańsk, Poland; 9 Infant Jesus Hospital, Medical University of Warsaw, 02-005 Warsaw, Poland

**Keywords:** Bacteriophage, Antibiotic, Phage–antibiotic synergy, Animal model, Human phage therapy

## Abstract

Bacteriophages (phages) may be used as an alternative to antibiotic therapy for combating infections caused by multidrug-resistant bacteria. In the last decades, there have been studies concerning the use of phages and antibiotics separately or in combination both in animal models as well as in humans. The phenomenon of phage–antibiotic synergy, in which antibiotics may induce the production of phages by bacterial hosts has been observed. The potential mechanisms of phage and antibiotic synergy was presented in this paper. Studies of a biofilm model showed that a combination of phages with antibiotics may increase removal of bacteria and sequential treatment, consisting of phage administration followed by an antibiotic, was most effective in eliminating biofilms. In vivo studies predominantly show the phenomenon of phage and antibiotic synergy. A few studies also describe antagonism or indifference between phages and antibiotics. Recent papers regarding the application of phages and antibiotics in patients with severe bacterial infections show the effectiveness of simultaneous treatment with both antimicrobials on the clinical outcome.

## Introduction

The growing degree of bacterial resistance to antibiotics has urged researchers to look for an alternative to antibiotic treatment such as among others phage therapy (PT) to treat different bacterial infections both in animals and humans [1–6]. Although there has been knowledge of this therapeutic method for over a hundred years there is still a substantial lack of randomized clinical trials that could, according to the current standards, confirm the efficacy of the application of bacterial viruses to combat bacterial infections. However, the results of many published case studies are promising. Thanks to the mechanism of antibacterial action being completely different from that of antibiotics, phages are able to lyse multidrug-resistant bacterial strains and have some other advantages over antibiotics. For example, they may amplify in the body or environment as long as host bacteria are present and even increase their load in the infection site, whereas the concentration of an antibiotic in the body declines over time. The selection of resistant mutants was lower for phages than for antibiotics [7]. However, some bacterial strains that became resistant to the phage regained sensitivity to antibiotics or turned out to be less virulent than the initial ones. The antibacterial range of phages is usually much lower than that of antibiotics, but this may potentially decrease the risk of PT on the natural microflora composition of the human body [2, 8]. The safety of the application of therapeutic phages may also be considered a significant benefit [9].

Phage preparations may be used alone or in combination with antibiotics, probiotics or synbiotics [2]. Using fewer antibiotics in an era of rising multidrug-resistant bacteria in favor of new alternative treatments, such as phage therapy seems promising. Moreover, recent research suggested [10–12] that the combined use of antibiotics and phages may yield much better results in combating bacterial infections. Phage–antibiotic synergy (PAS) is described as the interaction between two factors when the combined effect in a bacterial reduction is greater than the sum of either substance alone [13, 14]. Some advances in PAS research were presented in an article by Pirnay et al. [15]. Synergy of phage efficacy with antibiotics has been described in the literature [16]. Discussion of some clinical cases applying the synergy of phages and antibiotics was presented in an article by Segall [17]. Abedon et al. highlights that it is essential to note what antibiotics are applied along with PT and when those antibiotics are introduced to the treatment [18]. It is also important to determine the in vitro sensitivity of bacteria both to phages and antibiotics prior to their application. In silico findings show that combination therapy outperforms mono-treatments and its therapeutic effect is enhanced when interacting with the innate immune response [19].

This review is intended to present the studies and draw conclusions from research in vitro, in vivo and in clinical practice regarding the application of both phages and antibiotics in combating bacterial infections. The article emphasized the PAS observed in a group of phages against Gram-positive and Gram-negative bacteria including biofilms and the most important mechanisms of PAS for lytic and temperate phages as well as antagonism between phages and antibiotics. The potential for applying the combination of phages and antibiotics from in vivo studies in different branches of medicine was described. The application of phages and antibiotics in some case studies has been extended with recent literature from 2018 to 2022 and some perspectives of PAS in human medicine were presented.

## Phage–antibiotic synergy

Some antibiotics stimulate the production of phages by a bacterial host as well as form larger plaques in the presence of antibiotics [20, 21]. Also sublethal concentrations of certain antibiotics may enhance the release of progeny phages from bacterial cells [21, 22]. The existence of PAS can reduce the amount of antibiotics used in therapy and eventually decrease the emergence of antibiotic resistance in bacteria [23, 24]. Because the mechanism of killing bacteria differs between antibiotics and phages [13], researchers postulated that phages coupled with antibiotics may be more effective in controlling bacteria than alone [20].

Research indicates that the application of phages and antibiotics is particularly recommended for the treatment of Gram-positive bacteria including methicillin-resistant *Staphylococcus aureus* (MRSA) and multidrug-resistant *Enterococcus* strains. However PAS used to kill *S. aureus* was relatively more effective against the antibiotic-susceptible strain, than the antibiotic-resistant one [25]. This may suggest that antibiotic-induced alteration in bacterial membrane proteins that result in modification in the phage receptors [26, 27]. Simon et al. present the study of synergism between the lytic *S. aureus* phage Sb-1 at phage multiplicity of infection (MOIs) of 10^–1^ and 10 and oxacillin at concentrations ranging from 5 to 100 µg/ml for most examined *S. aureus* isolates [14]. A combination of phage Sb-1 and oxacillin caused a significantly stronger bacterial reduction than the antibiotic alone.

*Enterococcus* spp. isolates are leading causes of nosocomial infections with multi drug-resistant strains [28]. 80% and 90% of *Enterococcus faecium* strains are vancomycin- and ampicillin-resistant, thus infections are often treated with daptomycin (DAP). Limited studies have evaluated phage–antibiotic combinations against *E. faecium.* In one study, phage–antibiotic synergy including daptomycin was observed in a time-kill analysis and was associated with lower phage resistance. The next study by Morrisette et al. with the use of DAP and an *E. faecium* phage cocktail showed bactericidal activity in most regimens [29]. Moreover, DAP added to the phage prevented phage resistance against DAP resistant *E. faecium*.

The results from studies in vitro recommend the use of a combination of phages and some antibiotics against Gram-negative bacteria e.g., *Burkholderia cepacia*, *Pseudomonas aeruginosa* or *Citrobacter* spp., *B. cepacia* phages were examined for PAS in combination with meropenem, ciprofloxacin and tetracycline. Larger plaques and increased phage titres were observed when using increasing antibiotic concentrations. Moreover, the *B. cepacia* phage and low-dose meropenem when applied together increased the survival rate of *Galleria mellonella* larvae [21]. A study with a combination of phages and antibiotics against the *P. aeruginosa* strain PA#14 isolated from a burn was described by Aghaee et al. [11]. The *P. aeruginosa* strain was treated with a single phage, a mixture of two phages, and a combination of phages and antibiotics at a sub-minimum inhibitory concentration (MIC) and MIC levels. Four lytic phages were selected based on their performance in an initial efficiency of plating (EOP) test. Phages with distinct genetic features and infection properties out of the four initially sequenced were chosen. All of the selected phages were able to form plaques on *P. aeruginosa*. The results indicated that a combination of two phages and one antibiotic had the highest killing efficiency against the *P. aeruginosa* strain.

The study performed in vitro with *Citrobacter amalonaticus* showed synergistic effects for the use of phage MRM57 (10^3^ and 10^6^ plaque forming units (PFU/ml) with a sublethal dose of antibiotics with a different mechanism of action (carbenecillin, colistin, fosfomycin, gentamicin, meropenem, cefepime-tazobactam, tigecycline) except for cefotaxime at 1/10 × MIC [30].

## Possible mechanisms of PAS

**Fig. 1:**
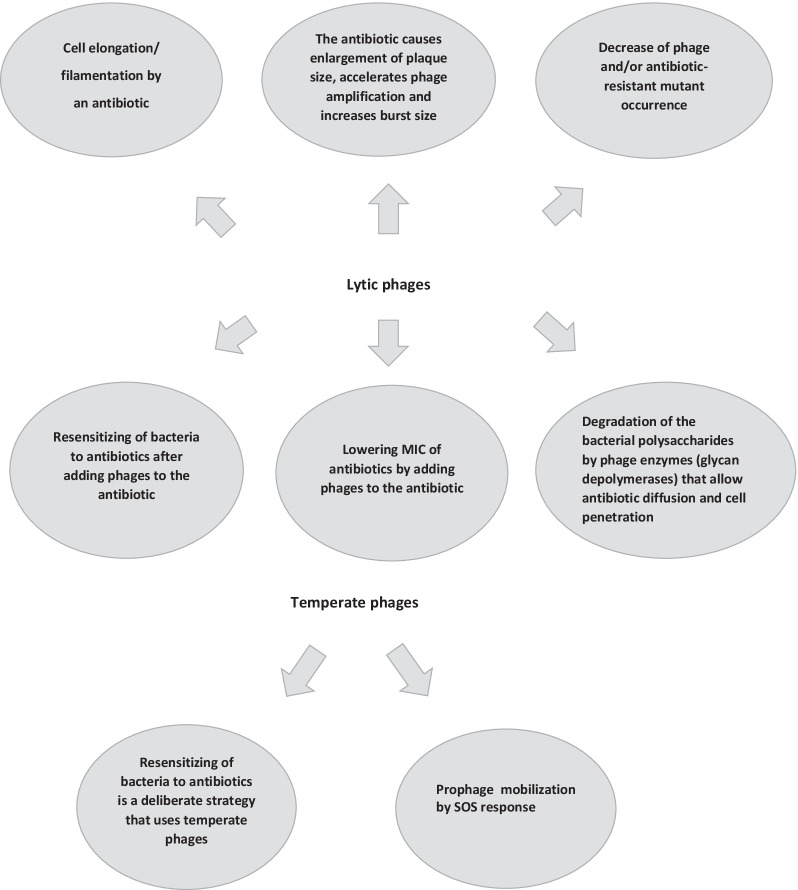
The most important PAS mechanisms leading to positive synergy

Different mechanisms can be suggested to explain the phenomenon of PAS: (1) cell elongation/filamentation by antibiotics; (2) increased plaque size by antibiotics, accelerated phage amplification and enhanced burst size; (3) decrease of phage and/or antibiotic-resistant mutant appearance; (4) increased antibiotic susceptibility due to the presence of the phage; (5) lowered MIC of antibiotics after adding phages to an antibiotic; (6) depolymerization of the bacterial polysaccharides by phage enzymes (glycan depolymerases) that increase antibiotic diffusion and cell penetration. The possible PAS mechanisms for lytic and temperate phages are shown in Fig. 1.

Comeau et al. described that cell filamentation is observed during the occurrence of the PAS phenomenon [10]. Beta-lactams and quinolones caused both filamentation and PAS in *Escherichia coli* and *Yersinia enterocolitica,* whereas gentamicin and tetracycline caused neither filamentation nor PAS. The authors suggest that some antibiotics may accelerate cell lysis given that filamentation induces perturbations in the peptidoglycan layer and this probably causes a greater sensitivity to the action of phage lysis genes encoded enzymes (e.g., endolysins, holins). As a result of this phenomenon, faster lysis and an increased rate of phage production may occur. The relationship between cell filamentation and PAS was also postulated by Knezevic et al. indicating that cell elongation/filamentation appears to be a necessary, but insufficient reason for PAS [31]. In this study, both ceftriaxone and ciprofloxacin caused cell enlargement. Only a subinhibitory concentration of ceftriaxone with a *Siphoviridae* phage σ − 1 against *P. aeruginosa* indicated PAS, but not with the *Podoviridae* phage δ and *Siphoviridae* 001A. Ceftriaxone inhibits cell wall synthesis, whereas phage amplification is not dependent on peptidoglycan synthesis. It was also highlighted that some antibiotics can disturb phage amplification by inhibition of DNA gyrase activity or protein synthesis. The synergy between the phage and antibiotic also depends on phage–host and phage–antibiotic combinations. The currently available data indicate that cell elongation/filamentation is one of the factors behind PAS, but there are other mechanisms involved. Indeed, synergy has also been observed with antibiotics that do not cause cell filamentation.

Another mechanism of PAS is the effect of the antibiotic that may lead to increased plaque size, faster phage amplification and/or enhanced burst size [32]. It was indicated that sublethal concentrations of linezolid, tetracycline and ketolide antibiotics can cause a 3-times increase in the plaque size of the *S. aureus Myoviridae* phage MR-5, whereas beta-lactam and quinolone antibiotics did not cause this effect [33]. An in vitro study demonstrated that a combination of a phage that infects *S. aureus* and antibiotics (clarithromycin, linezolid, cefotaxime, tetracycline and ciprofloxacin) increased the production of progeny phage [12].

PAS also increased the burst size of the T4 phage along with increasing cefotaxime concentrations [32]. The addition of 0.000186 and 0.00743 µg/ml of cefotaxime resulted in an increase in the T4 phage burst size from 8 to 80 and from 8 to 163 PFU/ml, respectively. Increasing the dose of antibiotics caused an increase in the burst size of the phage. With increasing concentrations of cefotaxime, phage concentration continuously increases to reach a maximum, with more than a 5-log increase in phage concentration, at 0.0625 µg/ml of cefotaxime, which was determined to be the optimal synergistic antibiotic concentration. Effects on the lytic cycle were also observed in the reduced latent period [32].

The next mechanism of PAS involves a reduction in the number of bacterial mutants resistant to phages and/or antibiotics. Interestingly, Oechslin et al. indicated that subinhibitory concentrations of meropenem and ciprofloxacin completely inhibit the occurrence of phage resistant mutants [34]. PAS was also observed in studies by Kebriaei et al. against MRSA strains, where *S. aureus* phage Sb-1-daptomycin/vancomycin combinations were superior over antibiotics alone and prevented the development of phage resistance [35]. Other in vitro studies showed that the number of phage resistant cells was smaller when PAS was observed [28, 36]. PAS, as a promising mechanism, was described by Li et al. as a combination of phages and antibiotics that reduces the dose of antibiotics and development of antibiotic resistance [22]. PAS refers to an increase in phage production after using sublethal levels of bactericidal antibiotics. Moreover, the *S. aureus* phage SA5 in combination with antibiotics reduces antibiotic resistance.

PAS can also be manifested as the sensitization of pathogens to approved antibiotics. Resensitizing bacteria to antibiotics was observed in an in vitro study with the use of a *P. aeruginosa* phage cocktail and antibiotics [37]. Treatment with ceftazidime, meropenem, gentamicin or ciprofloxacin in the presence of the *P. aeruginosa* phage cocktail PAM2H increased the number of *P. aeruginosa* bacteria susceptible to antibiotics by 63%, 56%, 31% and 81%, respectively. Most importantly, Wang et al. in an in vitro study with colistin and the *Acinetobacter baumannii Myoviridae* phage Phab24 observed that phage-resistant bacteria that evolved in the absence of antibiotics exhibited increased sensitivity to colistin, even though the antibiotic resistance mechanism remained unaltered [38]. This increase in antibiotic sensitivity is a direct consequence of the phage-resistance mechanism.

Resensitization of bacteria to antibiotics is a deliberate strategy that uses temperate phages. The other mechanisms refer to the fact that natural lysogens may be more susceptible to antibiotics due to their effect on the lytic cycle. The demonstration of synergy between temperate phages and antibiotics was presented by Al-Anany et al. [39]. In this study, temperate phage *E. coli* HK97 synergizes with ciprofloxacin to eradicate *E. coli *in vitro. Interestingly, the mechanism of temperate phage–antibiotic synergy is distinct from lytic phage–antibiotic synergy. The antibiotic does not merely stimulate phage production but acts through the RecA protein, an element of bacterial SOS response. The observed phenomenon is driven by depletion of lysogens. Interestingly, some antibiotics, like fluoroquinolones, may activate the lytic cycle in lysogenic bacteria.

The synergy of phages and antibiotics could also be due to a decrease in the MIC of antibiotics (e.g., amikacin, fosfomycin) [36, 40]. Another study indicated the in vitro synergistic activity of the *S. aureus Siphoviridae* phage Henu2 with sub-lethal concentrations of antibiotics on the decrease of *S. aureus* more than three logs within 12 h [12]. Phage Henu 2 alone exhibited weak inhibitory activity on *S. aureus* growth. The study showed that the combination of phage Henu2 and antibiotics increased the production of phages.

Combined therapies of phages and antibiotics were also examined in vitro to combat drug-resistant uropathogens [40]. A low dose of cefotaxime distinctly increased the production of phage ϕMFP by a uropathogenic *E. coli* strain [10]. A similar effect was observed for T4-like phages and beta-lactam and quinolone antibiotics and mitomycin C. Synergistic effects of the *E. coli* phage cocktail with antibiotics was shown by lowering MIC values of antibiotics [40]. The median MIC of amikacin was reduced from 8 to 2 µg/ml when the amikacin-phage cocktail combination was used. Similarly, the median MIC of fosfomycin was reduced from 32 to 8 µg/ml in a combination of phage with the antibiotic. It turned out that under certain conditions, phages provide an adjuvating effect by lowering the MIC for drug-resistant *E. coli* strains [36].

Another mechanism of PAS is related to the action of phage depolymerases. Among bacteriophages, depolymerases can be divided into peptidoglycan hydrolases, endorhamnosidases, alginate lyases, endosialidases and hyaluronate lyases [41, 42]. Phage depolymerases can occur in two forms: as a component of a virion particle, most often in the form of tail spikes or fibre proteins attached to the base plate, though they may also be located in other positions, and as a soluble protein generated during host lysis after phage maturation [42]. Polysaccharide depolymerases encoded by phages can specifically degrade bacterial structural polysaccharides (lipopolysaccharide LPS, peptidoglycan PG) or capsular polysaccharides, including exopolysaccharide compounds (EPS) in bacterial biofilms. These exopolysaccharides play important roles in maintaining the integrity of bacteria and bacterial virulence [41]. Depolymerases degrading EPS facilitate phage penetration and infection of biofilm-inhabiting bacteria [42–45]. Although very specific, depolymerases may have a broader activity than their parent phages, but it is suggested that multiple depolymerases are required for treating mixed biofilms [44]. Depolymerases can contribute to the PAS phenomenon as well, increasing antibiotic diffusion and facilitating cell penetration [41]. For example, alginate lyase derived from *Pseudomonas* phages can facilitate the diffusion of aminoglycosides to inhibit the growth of *P. aeruginosa* [46] or effectively eradicate *P. aeruginosa* biofilms [47].

## Antagonism between phages and antibiotics

One of the main reasons to study interactions between antibiotics and phages is to identify the existence of synergy so that it can be used to fight antibiotic-resistant bacteria. However, studies have shown that apart from PAS, phage–antibiotic antagonism can also be observed in some cases as a decreased efficacy of treatment compared to one of the individual treatments.

Some results indicate that a combination of specific phages and low-dose antibiotic treatments can actually cause antagonistic interactions between the bacteriophage and antibiotic. In Ali et al. we see examples of phage–antibiotic antagonism that were neglected as their focus was on the synergy of this combination [48]. However, when they combined their *S. aureus* isolate 7 with an MOI of bacteriophage and either ¼ × MIC of vancomycin or gentamicin, they obtained growth when there was no growth when this isolate was subjected only to an MOI of bacteriophage or an antibiotic was used concomitantly at a higher concentration of ½ × MIC. There was no further investigation into the mechanism behind the phage antibiotic antagonism and into what could be causing this growth.

Chaudhry et al. also report on interactions between phages and antibiotics within *P. aeruginosa* biofilms, experiencing phage antibiotic antagonism with phages and high levels of tobramycin [13]. Prior to combination with phages, high levels of tobramycin, 8 × MIC, decreased biofilm density more than low levels, 1 × MIC. However, upon combination with phages, the efficacy of the high level of tobramycin and bacteriophage treatment was less than that of the original 8 × MIC treatment. The combination of 1 × MIC tobramycin and phages was much more successful. Possible reasons cited for this phenomenon are tobramycin’s inhibition of phage replication at a high concentration, or even the antibiotic reducing the bacterial cell density to a point where the bacteriophage has trouble replicating. Bacteriophage replication is a cell density-dependent process; requiring bacterial cells to infect and replicate within, as well as additional nearby cells for its progeny to continue the infection cycle. A minimum density of bacterial cells is required for this bacteriophage replication to occur, known as the proliferation threshold [49]. If antibiotics lowered the density of bacterial cells below such a proliferation threshold before application of the phage, the bacteriophage would likely prove ineffective at replication due to its inability to expand through the bacterial population.

Another proposed mechanism for this phage–antibiotic antagonistic reaction can result from antibiotic interference with phage replication via inhibition of cell factors needed for this process, such as DNA gyrases or ribosomes. Tobramycin is a class of antibiotics known to bind to and inhibit ribosome function. If these ribosomes were needed for phage replication, this could also explain the decreased efficacy of the 8 × MIC tobramycin and phage treatment. Additional studies show that with the use of nalidixic acid and novobiocin, which inhibit DNA gyrase subunits A and B, respectively the synthesis of *E. coli* phages phi X174 and T5, as well as *Bacillus subtilis* bacteriophage SPO1, were inhibited [50–52]. Phage dependence on host proteins for replication is a case variable depending on the bacteriophage and the genes it encodes. For example, bacteriophage T4 is not dependent on *E. coli*’s DNA gyrase as it encodes its own topoisomerase and is able to use this for DNA unravelling during the phage replication cycle. Therefore, the inhibition of bacteriophage T4 replication by antibiotics nalidixic acid and novobiocin would be less than that of the reaction between the antibiotics and a bacteriophage such as T5 which is dependent on the host for its DNA gyrase [52].

Another mechanism refers to the antagonism of rifampicin with phage activity, which is related to the inhibition of bacterial RNA polymerase (RNAP) and phage transcription by rifampicin, while phages that carry their own RNA polymerase are not susceptible to treatment with rifampicin [53].

Recently, antagonistic interactions were observed between the polyvalent *Myoviridae* phage SaP7 infecting *Salmonella* and *E. coli* strains and several β-lactam antibiotics, e.g., amoxicillin/potassium clavulanate in piglet models and amoxicillin in mice models [54].

To date, there is little research available concerning antagonistic interactions between phages and antibiotics, however, there is evidence showing that this phenomenon does exist. Further investigation into phage antibiotic antagonism is paramount to understand the most effective clinical bacteriophage therapy.

## Phages and antibiotics in bacterial biofilms

Biofilm formation leads to corrosion and biofouling of industrial equipment [55]. It can also be the cause of many illnesses and infections in humans, such as oral diseases, native valve endocarditis, and a number of nosocomial infections [56]. Biofilms also play a role in the treatment delay of chronic wounds [57]. Biofilm formation is the first step to catheter-associated urinary tract infection (CAUTI) pathogenesis [58].

Studies on the application of phages and antibiotics against biofilms formed by Gram-negative bacteria are promising. After addition of cefotaxime and the T4 phage, especially at a high titre (10^7^ PFU/ml), to an *E. coli* biofilm, the minimum biofilm eradication concentration (MBEC) value decreased, suggesting the involvement of PAS in the complete eradication of *E. coli* biofilms in vitro [32]. The addition of low (10^4^ PFU/ml) and high (10^7^ PFU/ml) phage titres reduced the MBEC of cefotaxime against *E. coli* biofilms from 256 to 128 and 32 µg/ml, respectively. PAS was observed by increasing sublethal concentrations of cefotaxime resulting in an enhancement in T4 plaque size and T4 concentration [32]. Moreover, using bacteriophages and antibiotics individually to reduce biofilms often results in the emergence of significant levels of phage and antibiotic-resistant cells. Combining the T4 phage with tobramycin to weaken *E. coli* biofilms resulted in a greater than 99% and 39% reduction in antibiotic and phage resistant cells, respectively [24]. In *P. aeruginosa* biofilms, a combined therapy resulted in a 60% and 99% reduction in antibiotic and PB-1 phage resistant cells, respectively. Combined treatment was also more effective for the eradication of *Pseudomonas* bacteria in biofilms on cultured epithelial cells [13]. Phages can decrease the ascent of minority populations resistant to the treating antibiotic. Researchers reported that phages in combination with ciprofloxacin may increase the eradication of *Klebsiella pneumoniae* present in biofilms and stop the appearance of resistant variants [59, 60].

Phages and antibiotics used in the treatment of biofilms are more effective combined, due to the fact that their mechanisms of action complement each other. Phages can adhere to the specific receptors and penetrate biofilm layers through the pores and channels, thereby destroying the biofilm matrix [43]. In a biofilm, the individual bacterial cells are enclosed in a matrix of extracellular polymeric substances [61]. In some cases, bacteriophage tail spikes have depolymerase activity, which might be the reason why it can degrade EPS [62], and by that help the bacteriophage to penetrate the biofilm matrix and infect the bacterial cells [63]. Phages can reach and adsorb to cells in different biofilm layers, including the basal layer of the biofilm, causing the death of the cells [64]. Due to the destruction of the biofilm matrix, the bacterial cells were released as planktonic cells and then attacked by both antibiotics and phages. This may explain why taking advantage of PAS to kill bacterial cells forming biofilms is more efficient than used alone [64]. Studies on *Proteus mirabilis* have shown that the simultaneous use of ampicillin and phage vB_PmiS-TH has the greatest effect of reducing both planktonic and biofilm-forming bacteria [65]. The highest synergistic effect in the case of biofilms was found for the highest used ampicillin concentration 246 µg/ml and MOI of 100. In contrast, the highest synergy observed for planktonic bacteria was with an MOI of 1 or 0.001 and with the used antibiotic concentration of 8 µg/ml. Younger biofilms are definitely easier to eliminate, but the old biofilms cannot always be eliminated using antibiotics alone [60, 66, 67]. The combined therapy of phages and amoxicillin showed, in an 8-day-old biofilm, a significant log reduction of 5.5 in sessile cells compared to a log reduction of 3.5 and 3 caused by the bacteriophage at MOI of 0.01 or the drug at a higher concentration [67]. Overall, combined therapy is a more effective way to reduce older biofilms than using phages and antibiotics alone [67]. It is known that bacteriophage cocktails that target different host receptors delay the appearance of phage-resistant bacteria [68, 69]. Phage cocktails also enhance lytic effects by extending the phage host range [69]. A cocktail of phages in combination with antibiotics was proposed to treat biofilms in the human urine model [70]. A cocktail of phages against multidrug-resistant (MDR) *A. baumannii* with high lytic activity was used. The study demonstrated that some antibiotics commonly used in the treatment of urinary tract infections act synergistically with phage cocktails to reduce biofilm biomass.

Studies with *S. aureus* biofilms indicated synergy between phages and antibiotics. Adding phage SAP 26 to a 1-day-old biofilm formed by *S. aureus* D43 resulted in approximately 28% of bacterial cells being killed [64]. The combination of rifampicin with a phage showed the best biofilm removal effect (65% cells being killed) compared to the phage or antibiotic alone. Łusiak-Szelachowska et al. and Melo et al. described that the strategy of combining phages with antibiotics improves antibiofilm properties [71, 72]. Some antibiotics were more effective at lower doses in combination with phages. The sequence of application of phages and antibiotics in the elimination of biofilms may be important. This phenomenon is confirmed by research of the biofilm formed by the *S. aureus* strain ATCC 35556, which was treated with the phage SATA-8505 and antibiotics: tetracycline, vancomycin, linezolid, cefazolin and dicloxacilin using various treatment strategies [73]. A significant reduction in the bacterial load was observed when the phage was added before antibiotic treatment, especially for vancomycin and cefazolin at lower concentrations. The results of this research show that phages can augment antibiotic activity. The sequence of application of phages and antibiotics was also tested in mono- and dual-species biofilms with the *P. aeruginosa Myoviridae* phage EPA1 and seven antibiotics (erythromycin, tetracycline, meropenem, chloramphenicol, gentamicin, ciprofloxacin, and kanamycin) [74]. The introduction of sequential treatment, consisting of phage administration followed by an antibiotic (gentamicin or ciprofloxacin), improved the effects of therapy against a *P. aeruginosa* mono-species biofilm. As a result of the tests, it can be concluded that a sequential administration of phages and antibiotics in the treatment of biofilms can bring the best results. The concentration of phages and antibiotics and the time of antibiotic application are essential factors when considering combined treatments. Studies with a combination of phages and some antibiotics indicated that this type of treatment may effectively eliminate biofilms formed by both Gram-positive and Gram-negative bacteria.

## Phages and antibiotics in animal models

Phage–antibiotic synergy was tested using a wide variety of antibiotics in different animal models in vivo or ex vivo in research conducted on chicken, mice, and rats (Table 1). The results may be very helpful to assess the potential of phage and antibiotic application in different branches of medicine.

**Table 1:** Bacteriophage and antibiotic combination in in vivo studies

In vivo model					
Model	Antibiotic	Bacterial strain	Phage	Effect	References
BALB/c mice (intraperitoneally injection)	Gentamicin (0.8 mg/kg)	*P. aeruginosa* K (PAK) 10^7^ CFU/ml injected intraperitoneally	*P. aeruginosa* Pf1 (filamentous) 3 × 10^10^ PFU	Synergy	Hagens et al. [75]
BALB/c mice (intravenous injection—sepsis)	Clindamycin (8 mg/kg)	*S. aureus* MDRSA 10^8^ CFU/ml injected intravenously	*S. aureus* (lytic) 10^8^ PFU/ml MOI of 1	Treatment with the phage was more effective than with clindamycin or combination treatment	Oduor et al. [77]
Diabetic BALB/c mice (Hindpaw infection)	Linezolid (25 mg/kg)	*S. aureus* 43300 (MRSA) 10^4^ CFU/10 µl	*S. aureus* MR-10 (lytic) 10^8^ PFU/ml MOI of 100	Synergy	Chhibber et al. [78]
A murine air pouch model of infection	Linezolid 2.5 mg/kg	*S. aureus* ATCC 43300 MRSA 0.1 ml of 10^5^ CFU/ml	*S. aureus* MR-5 0.1 ml of 10^6^ PFU/ml	Synergy	Kaur and Chhibber [79]
Mice dorsal wound model	Ceftazidime (CAZ) 410 mg/kg of CAZ at 5 µl/g	*P. aeruginosa* PAO1::lux 1 × 10^7^ CFU	*P. aeruginosa* phage cocktail PAM2H 25 µl of 1 × 10^8^ PFU	Synergy	Engeman et al. [37]
Osteomyelitis model in rats	Teicoplanin 20 mg/kg/day	*S. aureus* 0.05 ml of 5 × 10^5^ CFU/ml	*S. aureus* Sb-1 0.1 ml of 3 × 10^7^ PFU	Synergy	Yilmaz et al. [80]
Osteomyelitis model in rats	Imipenem + Cilastatin 120 mg/kg/day Amikacin 25 mg/kg/day	*P. aeruginosa* 0.05 ml of 5 × 10^5^ CFU/ml	*P. aeruginosa* PAT14 0.1 ml of 3 × 10^7^ PFU	Synergy	Yilmaz et al. [80]
Mice with post arthroplasty model of infection	Linezolid 5% w/w mixed with Hydroxypropylmethylcellulose (HPMC) gel as the biopolymer	*S. aureus* MRSA 43300 10 µl of 10^6^ CFU/ml	*S. aureus* MR-5 10^9^ PFU/ml mixed with HPMC gel as the biopolymer	Synergy	Kaur et al. [81]
BALB/c mice nasal infection	Mupirocin (5 mg/kg)	*S. aureus* 43300 (MRSA) 10^6^ CFU/ml	*S. aureus* MR-10 (lytic) 50 µl of 10^7^ PFU/ml	Synergy	Chhibber et al. [82]
Colibacilosis in chicken	Enrofloxacin (50 ppm for 7 days)	*E. coli* 0.1 ml of 6 × 10^5^ CFU/ml	*E. coli* phage cocktail DAF6 and SPR02 10^9^ PFU/ml	Synergy	Huff et al. [83]
Mice with acute pneumonia	Gentamicin 1.5 mg/kg	*K. pneumoniae* W-KP2 (K47 serotype) 1 × 10^9^ CFU	*K. pneumoniae* P-KP2 1 × 10^9^ PFU	Synergy	Wang et al. [84]
Neutropenic mouse model of acute lung infection in mice	Ciprofloxacin 0.33 mg/mg	*P. aeruginosa *FADD1-PAOO1 25 µl of bacteria (approx. 10^6^ cells)	*Pseudomonas* PEV20 10^6^ PFU/mg	Synergy	Lin et al. [85]
Methicillin-resistant pneumonia in rats	Linezolid 10 mg/kg	*S. aureus* AW7 1 × 10^10^ CFU	*S. aureus* phage cocktail 3 × 10^10^ PFU	Linezolid and aerophages did not synergize	Prazak et al. [86]
Methicillin-resistant pneumonia in rats	Daptomycin 6 mg/kg	*S. aureus* AW7 1 × 10^10^ CFU	*S. aureus* phage cocktail 2 × 10^10^ PFU	Simultaneo-us treatment was not more effective than aerophage therapy	Valente et al. [87]

CAZ: ceftazidime; CFU: colony-forming unit; HPMC: Hydroxypropylmethylcellulose; MDRSA: multidrug-resistant *S. aureus*; MOI: phage multiplicity of infection; MRSA: methicillin-resistant *S. aureus*; PFU: plaque-forming unit

### Intensive care/systemic infections (e.g. sepsis)

The synergism between the *P. aeruginosa Inoviridae* phage Pf1 and gentamicin was studied in vivo in mice [75]. Mice were injected intraperitoneally with *P. aeruginosa* K-PAK with either gentamicin (0.8 mg/kg), or the *P. aeruginosa* phage Pf1 (3 × 10^10^ PFU) or a combination of the antibiotic and the phage. The control group and the group treated with the antibiotic alone died within 24 h following the bacterial challenge. Mice treated with the phage alone died within 48 h, however, the combined therapy rescued more than 70% of the mice. Moreover, an in vitro study in this research proved that bacteria harbouring a plasmid carrying several antibiotic resistant genes (e.g., to tetracycline), when treated with a filamentous phage exhibited lowered resistance to these antibiotics (here, to tetracycline). These results suggest possible resensitisation of bacteria to the antibiotic when bacteria is treated with phage together with antibiotic. The study indicated the occurrence of synergy between the used phage and the antibiotic [75]. Recently, the synergism of the *S. aureus* phage cocktail and a low-dose standard of care flucloxacillin was confirmed in a rodent model of experimental endocarditis. However, the antibiotic partially suppressed in vivo phage replication [76].

A study that may suggest the existence of antagonism between the *S. aureus* phage and clindamycin was performed [77]. A combination treatment of clindamycin (8 mg/kg) with the lytic *S. aureus* phage (10^8^ PFU/ml) was compared with mono-therapy of either antibiotics or phages on mice with sepsis. The in vivo experiment involved infection by the multidrug-resistant *S. aureus* MDRSA strain injected intravenously (iv) via a tail vein with treatments done at either 24 h or 72 h post-infection. Phage mono therapy had 100% eradication against bacterial infection at both observation intervals, whereas the antibiotic yielded 62% and 87% eradication at 24 h and 72 h respectively. The combined treatment proved to be 75% and 90% eradication at 24 h and 72 h post-infection [77]. Phage-clindamycin efficacy was dependent on time; by day 10, a few bacteria in the blood were detected. The result of this study suggests that not all antibiotics may work efficiently when used in co-therapy.

### Surgery/wounds and soft tissue infections

Synergism was investigated between the *S. aureus* phage MR-10 and linezolid in a hindpaw infection caused by the methicillin-resistant *S. aureus* 43300 strain in diabetic mice [78]. The *S. aureus Myoviridae* phage MR-10 (10^8^ PFU/ml) and linezolid (25 mg/kg) were tested alone as well as in combination. In the combined therapy group, tissue healing was hastened and the oedema levels were significantly lower. A comparison between bacterial loads taken from each (mono-, or combined therapy) group showed that the combined therapy was maximally efficient at reducing the bacterial load, which suggests the existence of synergy between the phage and antibiotic [78]. Another study of synergy between phages and antibiotics was performed in a murine air pouch model of infection mimicking skin and soft tissue infection using the *S. aureus* MR-5 phage and linezolid [79]. Experimental skin infection was induced by *S. aureus* ATCC 43300 (MRSA) and the *S. aureus* MR-5 phage was administered subcutaneously 0.1 ml 10^6^ PFU/ml alone or with linezolid administered orally at dose 2.5 mg/kg. The combination of both factors showed synergy in this model of infection [79]. Other studies in vivo in a mouse dorsal wound model with infected mice *P. aeruginosa* demonstrated that 7 out of 8 mice treated with ceftazidime (CAZ) 410 mg/kg at 5 µl/g body weight and the *P. aeruginosa* phage cocktail PAM2H 25 µl of 1 × 10^8^ PFU for 3 days had no detectable bacteria in wounds on day 4 [37]. All mice treated with the antibacterial factor alone had ~ 10^7^ colony forming units (CFU) in wounds. Treatment with combination of phages and antibiotics resulted in a synergistic reduction of bacterial burden in vivo.

### Orthopedics/orthopedic infections/peri-implant infections

Studies in vivo in an osteomyelitis model in rats demonstrated that simultaneous application of the *S. aureus* Sb-1 or *P. aeruginosa* PAT14 phages and antibiotics significantly increases the elimination of biofilms [80]. An implant-related osteomyelitis infection model in rats was treated locally with phages (0.1 ml containing 3 × 10^7^ PFUs) on 3 consecutive days, antibiotics for 14 days and a combination of phages and antibiotics. The MRSA infected rats were treated with the Sb-1 phage and teicoplanin 20 mg/kg/day, while the *P. aeruginosa* infected rats received the PAT14 phage and imipenem + cilastatin 120 mg/kg/day and amikacin 25 mg/kg/day. The MRSA biofilm was significantly eliminated in the group of simultaneous treatment with the antibiotic and phage. The strongest significant reduction of *P. aeruginosa* in the group of simultaneous treatment with the antibiotic and phage was observed. In conclusion, a synergistic effect of phages and different antibiotics was observed in vivo in the treatment of *S. aureus* and *P. aeruginosa* biofilms [80]. The next study of synergy between phages and antibiotics with a combination of the *S. aureus* phage MR-5 and linezolid was examined in mice with a post arthroplasty model of the *S. aureus* infection [81]. Mice were implanted with a wire coated with the *S. aureus* MR-5 phage 10^9^ PFU/ml and/or linezolid 5% into the intra-medullary canal of the femur bone followed by inoculation of *S.* *aureus* MRSA. The bacterial burden in the control in the surrounding joint tissue was detected on day 5, indicating ~ 8 log CFU. In the group of phages or the combination of phages and linezolid coated wires, the bacterial burden in tissue had a significant reduction of > 3 logs and of 4.5 logs from days 5 and 7 and sterile tissue by day 10. Studies demonstrated no appearance of resistant mutants in any of the phage and/or linezolid implanted mice. These studies in vivo of orthopedic-related infections indicated synergy between the phage and antibiotic.

### Laryngology/upper respiratory tract infections

The *S. aureus* phage MR-10 and mupirocin were studied to confirm the effectiveness of this dual treatment in a mouse nasal infection of *S. aureus*, where the 43300 strain (MRSA) was administered intranasally [82]. Mupirocin (5 mg/kg) and the *S. aureus* phage MR-10 (50 µl of 10^7^ PFU/ml) were provided intranasally the next day. Ex vivo tests on murine nasal epithelial cells (NEC) examined nasal bacterial load in tissue and revealed that the combined therapy managed to completely clear bacteria by day 5 after infection. In comparison, mono-therapies took 7 days to significantly reduce bacterial load, such that by day 10 post-treatment no bacteria were found in the samples. Moreover, research has indicated that the frequency of emergence of spontaneous mupirocin-resistant mutants was dropped to negligible levels when a combined approach was used. The results of this study clearly show that the dual approach of mupirocin and phages is superior to mono-therapies when fighting *S. aureus* MRSA nasal infection.

### Pulmonology/lower respiratory tract infections

The synergy between enrofloxacin and an *E. coli* phage cocktail was examined in an in vivo model of respiratory infection on chickens [83]. Injected *E. coli* caused severe airsacculitis of the animals. Chickens were treated with enrofloxacin alone (in drinking water 50 ppm for 7 days), with the *E. coli* phage cocktail alone (single injection—10^9^ PFU/ml) or a combination of these two. Mono-treatments of phages and antibiotics resulted in 15% and 3% mortality of birds respectively. The research concluded that the best result on the course of infection was in the combined therapy group, where birds were completely protected from the infection [83]. The other study of synergy of a combined treatment with the *K. pneumoniae* phage P-KP2 and gentamicin in mice vs. mono-treatments with acute pneumonia caused by *K. pneumoniae* W-KP2 (K47 serotype) was demonstrated by Wang et al. [84]. Mice were infected intranasally with bacteria and 1 h post-infection were treated intranasally with the phage 1 × 10^9^ PFU and 30 min after phage administration gentamicin at dose 1.5 mg/kg was administered. Pneumonia symptoms in mono-treatments were significantly alleviated and the survival rate increased to 70%. That the combined treatment of phages and gentamicin completely rescued infected mice suggests the existence of synergy between the phage and the antibiotic [84]. The synergisitic effect was also investigated in a neutropenic mouse model of acute lung infection when using ciprofloxacin 0.33 mg/mg and the *Pseudomonas* phage PEV20 (10^6^ PFU/mg) in powder [85]. Mice were infected with *P. aeruginosa* FADD1-PAOO1. The combination of PEV20 and ciprofloxacin powder significantly decreased the bacterial load in mouse lungs by 5.9 logs, whereas no obvious reduction of bacteria was observed in mice in mono-treatments. The study in vivo demonstrated the synergistic effect of PEV20 and ciprofloxacin [85].

However, a study of a combined treatment of an *S. aureus* phage cocktail and linezolid on a rat model of methicillin-resistant pneumonia caused by *S. aureus* (MRSA) AW7 indicated indifference [86]. The effects of the *S. aureus* phage cocktail (3 × 10^10^ PFU) in various forms were investigated: intravenous, aerosol, a combination of intravenous phages with phage ventilation, and a combination of aerophages with linezolid 10 mg/kg [86]. Of course, there were also studies using linezolid alone. The results showed that the best treatment option for MRSA pneumonia was a combination of intravenous and ventilation phages, which saved 91% of the rats subjected to such therapy. Moreover, the use of aerosolized phages alone or the intravenous phage allowed 50% of the tested rats to survive. Intravenous linezolid alone reduced mortality in 38% of the rats. It did not act synergistically with aerophages (55% survival). Moreover, in vitro studies have shown that such a combination could be harmful (abolition of phage amplification) [86]. Also the effect of indifference of systemic daptomycin and a nebulized *S. aureus* phage cocktail on the treatment of MRSA pneumonia in vivo in rats was indicated [87]. Ventilator-associated pneumonia caused by the MRSA clinical strain AW7 was treated with simultaneous application of intravenous daptomycin 6 mg/kg and the nebulized *S. aureus* phage cocktail (2 × 10^10^ PFU) or only with aerophages. The simultaneous treatment of antibiotics and phages was not more effective than aerophage therapy in the survival of rats, as well as bacterial burdens in the lungs or spleen. The survival of rats was respectively 55% vs. 50% after a 96-h trial [87].

In vivo studies in different animal models mainly showed synergy between some specific phages and antibiotics, particularly for models of systemic infections in mice, soft tissue infections in mice, orthopedic infections in mice and rats, as well as respiratory infections in mice and chickens. However, a few studies performed on a model of sepsis in mice and a model of pneumonia in rats indicated antagonism or indifference between some phages and antibiotics, respectively. The type of interaction may depend on the type and dose of the used antibiotic and phage as well as the time of administration. The studies suggest that the combination of some phages and antibiotics can be potentially used in different branches of medicine.

## Phages and antibiotics in human phage therapy

Studies on the combined action of phages and antibiotics in the fight against different bacterial infections in human medicine is the subject of research interest.

The use of phages or combinations of phages with antibiotics has also been studied in patients with suppurative bacterial infections in the 1980s in Poland [88–91]. Phages were administered orally or locally and more effective results were obtained for patients who used only phages (about 96% of positive cases) compared to a combined treatment with phages and antibiotics (about 85% of positive cases). The differences were statistically significant. The obtained results suggest the existence of antagonism between phages and antibiotics.

Zilistenau et al. also checked the efficacy of phage therapy or a combination of phage and antibiotic treatment in 87 patients with chronic urinary tract infections [92]. Phages were used orally at a dose of 20 ml for 5 consecutive days. Antibiotics were given for up to 10 days. The best efficacy was obtained after phage treatment in 92.8% of positive cases, while good results were obtained in 64.4% of patients using phages and antibiotics [92]. The treatment results for both groups of patients suggest the existence of antagonism between phages and antibiotics.

Recent experiences in human clinical therapy with administration of phages and antibiotics are presented in Table 2 [93–99]. The cases, presented in Table 2, treated with phages and antibiotics show the favorable influence of both factors on the course and outcome of the treatment. Prior to the use of phages and antibiotics, susceptibility testing of bacterial strains causing bacterial infections in patients was performed. In a recent case of clinical therapy with phages and antibiotics, 1 patient took part [93]. A post-operative 76-year-old patient with a chronic *P. aeruginosa* infection of an aortic Dacron graft with associated aorto-cutaneous fistula was treated with a single application of the *P. aeruginosa* phage and ceftazidime. The preparation was given to the patient for mediastinal fistula. The preparation consisted of 10 ml of the OMKO1 phage at a concentration of 10^7^ PFU/ml and a solution of 0.2 g/ml ceftazidime. Ceftazidime was continued at home. The patient was cured without any recurrence of infection [93].

**Table 2:** Phages and antibiotics in recent clinic experiences

Disease	Phage	Antibiotic	Clinical outcome	References
76-year-old patient with a chronic *P. aeruginosa* infection of an aortic Dacron graft with associated aorto-cutaneous fistula	Single application 10 ml of the OMKO1 phage at a concentration of 10^7^ PFU/ml for mediastinal fistula	Before phage therapy (PT) prolonged the course of antibiotics Along with PT 0.2 g/ml ceftazidime was administered for mediastinal fistula. Ceftazidime was continued at home	Approx. 4 weeks post-procedure, the patient developed significant bleeding from the mediastinal wound. Due to concerns that an aortic perforation may occur, the patient underwent exploratory surgery. The patient was cured without any recurrence of infection	Chan et al. [93]
42-year-old patient with a trauma-related left tibial infection with drug resistant *A. baumannii* and *K. pneumoniae*	Combination of phages: ϕAbKT21phi3 and ϕKpKT21phi1 1 ml of each phage 5 × 10^7^ PFU/ml administered intravenously (iv) 3-times daily (tid) over 35 min for 5 days. Second course: 6 days 1 week later	Before PT prolonged course of antibiotics Simultaneously with PT administered iv meropenem (2 gr tid) and colistin 4.5 × 10^6^ units/bid	Rapid tissue healing and positive culture eradication. The patient’s leg did not have to be amputated and he is undergoing rehabilitation	Nir-Paz et al. [94]
30-year-old patient with a fracture-related pandrug-resistant *K. pneumoniae*	Pre-adapted phage M1 (10^8^ PFU/ml) used locally (in the surgical wound via a catheter) for 6 days	After unsuccessful antibiotic therapy simultaneously with PT administered meropenem and colistin followed by ceftazidime/avibactam	Finally, clinical and microbiological improvement was observed	Eskenazi et al. [95]
26-year-old cystic fibrosis (CF) patient awaiting lung transplantation with multidrug resistant (MDR) *P. aeruginosa* pneumonia, respiratory and renal failure	Combination of 4 lytic phages *P. aeruginosa:* AB-PA01 4 × 10^9^ PFU/5 ml administered iv every 6 h for 8 weeks	Before PT prolonged course of antibiotics Simultaneously with PT systemic antibiotics: ciprofloxacin, piperacillin–tazobactam for 3 weeks. Later ciprofloxacin was discontinued and doripenem was added	Clinical resolution of infection, no recurrence of pneumonia and CF exacerbation within 100 days after PT. Successful bilateral lung transplantation 9 months later	Law et al. [96]
Three lung transplant recipients (LTR) with life-threatening MDR infections caused by *P. aeruginosa* (n = 2) and *Burkholderia dolosa* (n = 1). Two patients had *P. aeruginosa* pneumonia. A third patient had recurrent pneumonia *B. dolosa* infection following transplant	Case 1 received *P. aeruginosa* phage cocktail: AB-PA01 (10^9^ PFU/ml) and two phage cocktails (10^7^–10^9^ PFU/ml) administered iv and nebulized Case 2 received *P. aeruginosa* phage cocktail AB-PA01 (10^9^ PFU/ml) iv. Case 3 received single lytic phage BdPF16phi4281 (10^6^–10^7^ PFU/ml) iv PT was conducted for variable durations	Simultaneously with PT antibiotics Case 1: Post-transplant he had two episodes of MDR *P. aeruginosa* pneumonia Episode 1: systemic antibiotics (piperacillin–tazobactam and colistin Episode 2: Systemic antibiotics (piperacillin–tazobactam, tobramycin and inhaled colistin) Case 2: Post-transplant she had an MDR *P. aeruginosa* infection. Additionally *Mycobacterium abscessus* pulmonary infection treated with antibiotics Along with iv *P. aeruginosa* phage inhaled colistin. The isolate grown at day 60 and subsequent strains were successfully treated with piperacillin–tazobactam Case 3: At the time of lung transplant and after she received antibiotics. Then the phage was added to ceftazidime–avibactam and piperacillin–tazobactam. On week 10 of PT infusion meropenem, extended infusion ceftazidime–avibactam, minocycline, and inhaled tobramycin Her sepsis resolved, bloodstream infection cleared and her respiratory status improved. However, she developed progressive liver failure with concern for drug-induced toxicity, including from minocycline, dapsone, and posaconazole prophylaxis. She again developed pneumonia	Both patients with *P. aeruginosa* infection responded clinically and were discharged from the hospital off ventilator support. *B. dolosa* infection relapsed on PT and the patient expired	Aslam et al. [97]
52-year-old critically ill patient with MDR *A. baumannii* respiratory infection	*A.baumannii* phage AbW4878Ø1 iv. 1 × 10^9^ PFU/ml twice daily and nebulized 0.1 × 10^9^ PFU/ml twice daily along with antibiotics for a total 35 days	Before PT course of antibiotics. Along with PT broad-spectrum of antibiotics infusions	Successfully treated with antibiotics and intravenous and nebulized PT	Rao et al. [98]
63-year-old patient with a recurrent urinary tract infection caused by *K. pneumoniae*	*K. pneumoniae* phage cocktail after two rounds of PT causes appearance phage resistant mutants. Then combined use of phage and non-active antibiotics was used Phage cocktail III (5 × 10^8^ PFU/ml for each phage) was administered by bladder-irrigation once a day for 5 days	*K. pneumoniae* was completely resistant to sulfamethoxazole–trimethoprim Antibiotics (800–160 mg) was used along with PT orally twice a day for 5 days	Synergistic effect was observed. Phage and higher dose of antibiotics inhibited the emergence of phage resistant mutant in vitro. The patient was successfully cured by this combination	Bao et al. [99]

CF: cystic fibrosis; iv: intravenously; LTR: lung transplant recipients; MDR: multidrug-resistant; PFU: plaque-forming unit; PT: phage therapy, tid: 3-times daily

A successful combination of phages and antibiotics has also been used in orthopedic infections in humans [94, 95]. A 42-year-old patient with a trauma-related left tibial infection with drug-resistant *A. baumannii* and *K. pneumoniae* treated with a combination of the phages *A. baumannii* and *K. pneumoniae* administered intravenously for 5 days and 1 week later for 6 days with meropenem and colistin iv simultaneously had a good clinical outcome with rapid tissue healing and positive culture eradication [94]. Recently, a clinical case of a 30-year-old patient with a fracture-related pandrug-resistant *K. pneumoniae* was treated with phages and antibiotics [95]. Testing of a day-702 *K. pneumoniae* before PT indicated non-susceptibility to all antibiotics in all antimicrobial categories. After long-term antibiotic therapy, the patient was treated locally with the pre-adapted therapeutic phage M1 simultaneously with meropenem and colistin followed by ceftazidime/avibactam for 6 days. Importantly, the in vitro phage and antibiotics were highly effective against the infecting *K. pneumoniae* strain in suspensions and in biofilms. Clinical improvement was observed after combined treatment in this clinical case. Considering the clinical data and the in vitro phage–antibiotic synergy data, there is evidence that a combination of the phage M1 and the antibiotics meropenem and ceftazidime/avibactam finally caused clinical improvement in this patient [95].

An effective combined treatment with phages and antibiotics was used in respiratory infections in humans [96–99]. A 26-year-old cystic fibrosis (CF) patient with MDR *P. aeruginosa* pneumonia awaiting lung transplantation was treated with a *P. aeruginosa* phage cocktail AB-PA01 iv for 8 weeks and simultaneously for 3 weeks with systemic antibiotics: ciprofloxacin, piperacillin–tazobactam [96]. Later ciprofloxacin was discontinued and doripenem was added. No recurrence of pneumonia and CF exacerbation within 100 days after phage therapy was observed [96]. Another study with three lung transplant recipients with life-threatening MDR infections caused by *P. aeruginosa* (n = 2) and *Burkholderia dolosa* (n = 1) was reported by Aslam et al. [97]. Patients developed pneumonia, which was treated with the *P. aeruginosa* phage cocktail AB-PA01 iv or nebulized or single lytic phage *B. dolosa* iv with different antibiotics conducted for variable durations. Patients with the *P. aeruginosa* infection had good clinical outcomes and only patients with the *B. dolosa* infection relapsed on PT and the patient expired [97]. Another critically ill patient with an MDR *A. baumannii* respiratory infection was successfully treated with the *A. baumannii* phage AbW4878Ø1 iv. 1 × 10^9^ PFU/ml twice daily and nebulized 0.1 × 10^9^ PFU/ml twice daily along with broad-spectrum antibiotics for a total of 35 days [98]. The patient was efficiently treated with the combination of phages and antibiotics and was discharged and continued administration of eravacycline, meropenem and polymyxin B as a precautionary measure.

However, non-active antibiotics and bacteriophage synergism was observed in successfully treating a recurrent urinary tract infection caused by extensive drug-resistant *K. pneumoniae* [99]. A 63-year-old patient was treated with a *K. pneumoniae* phage cocktail in two rounds of PT with emergent phage resistant mutants. Then the *K. pneumoniae* phage cocktail was administered by bladder irrigation once a day and non-active sulfamethoxazole–trimethoprim orally twice a day for 5 days. Although, antibiotics used with PT were not active against bacterial isolates from the urine of the patient a synergistic effect for this combination was observed. What is more, the phage and higher doses of antibiotics inhibited the emergence of phage resistant mutants in vitro. The patient was successfully cured with this combination.

Nowadays, phages are used alone especially against antibiotic-resistant bacteria or as a supplement for existing antimicrobials to improve the effectiveness of therapy or where antibiotic therapy has repeatedly failed. The application of phages and antibiotics, including in recurrent bacterial infections in humans in recent years has shown promising treatment results. Good clinical results in studies from 2018 to 2022 with combined treatments in severe cases with orthopedic, respiratory and urinary tract infections were obtained. It has been proven that combined treatments in human medicine may increase the effectiveness of phages as well as antibiotics and may reduce phage resistance acquisition, which could be important in an era of antibiotic resistance. In the absence of randomized cilinical trials, phage–antibiotic interactions should be investigated in vitro to prevent antagonism and to confirm synergy to achieve better understanding results of treatments. Phage–antibiotic interactions may depend on the type and dose of each agent and the time of administration. More studies on the combined action of phages and antibiotics in vitro and in vivo should be carried out in the near future and studies in clinical trials are urgently needed.

## Conclusions

Studies on the combined action of phages and antibiotics is gaining importance especially now in the era of antibiotic resistance. The combination of phages and antibiotics investigated in in vitro studies may suggest a phenomenon of PAS, where antibiotics may increase the production of phages and where a decrease of phages and/or antibiotic-resistance has been observed. The main mechanisms of the PAS are also based on cell elongation/filamentation by antibiotics, enhancement of antibiotic susceptibility in the presence of the phage, decreased MIC of antibiotics after adding phages and depolymerization of the bacterial polysaccharides by phage glycan depolymerases that facilitates antibiotic diffusion and cell penetration. Antagonism between some phages and antibiotics in in vitro studies was also found, but less frequently. Antagonism or indifference were also found in a few in vivo studies. Although the research on antagonism is scarce, it is worth studying in vitro the interactions between phages and antibiotics to obtain better treatment results. Combinations of some phages and antibiotics showed synergy in some systemic infections, soft tissue infections, orthopedic or respiratory tract infections in vivo in animals. Recent clinical cases with administration of some specific phages and antibiotics to which the infecting bacteria were susceptible showed good clinical outcomes, e.g., in some orthopedic or respiratory tract infections. Further studies are needed to examine the efficacy of phage–antibiotic combinations, particularly in clinical settings. Those studies should specify which antibiotics and phages can be synergistic, what concentrations of both agents are optimal as well as the timing of application of both agents. Finally, clinical trials involving phage–antibiotic combinations should provide a definite answer as to the applicability of that approach.

## Data Availability

Not applicable.

## Abbreviations

CAZ: Ceftazidime
CF: Cystic fibrosis
CFU: Colony forming units
DAP: Daptomycin
EOP: Efficiency of plating
EPS: Exopolysaccharide
HPMC: Hydroxypropylmethylcellulose
iv: Intravenously
LPS: Lipopolysaccharide
LTR: Lung transplant recipients
MBEC: Minimum biofilm eradication concentration
MDR: Multidrug-resistant
MDRSA: Multidrug-resistant *S. aureus*
MIC: Minimum inhibitory concentration
MOI: Phage multiplicity of infection
MRSA: Methicillin-resistant *S. aureus*
NEC: Nasal epithelial cells
PAS: Phage–antibiotic synergy
PFU: Plaque forming units
PG: Peptidoglycan
PT: Phage therapy
RNAP: RNA polymerase
tid: 3-Times daily
